# The Weighing Chair of Sanctorius Sanctorius: A Replica

**DOI:** 10.1007/s00048-018-0193-z

**Published:** 2018-05-14

**Authors:** Teresa Hollerbach

**Affiliations:** Max-Planck-Institut für Wissenschaftsgeschichte, Boltzmannstraße 22, 14195 Berlin, Germany

**Keywords:** Early Modern History of Medicine, Sanctorius Sanctorius, Quantification, Replication Method, Physiological Research, Frühneuzeitliche Medizingeschichte, Sanctorius Sanctorius, Quantifizierung, Replikationsmethode, Physiologie

## Abstract

In 1614, the physician Sanctorius Sanctorius (1561–1636) published his most famous work entitled *Ars […] de statica medicina *(*On static medicine*). This is a work composed of aphorisms that present the practical results of a series of weighing procedures, rather than theoretical observations. *De statica medicina* is the result of a large number of test series that Sanctorius carried out over many years with the weighing chair he constructed himself in order to quantify the so-called *perspiratio insensibilis*, an insensible perspiration of the human body. Through his weighing experiments, Sanctorius introduced the idea of quantitative research into physiology. Although historical accounts ascribe an important role to Sanctorius as the founder of a new medical science, up until now the design of his weighing chair and the method of measurement have not been closely analysed. The aim of this paper is to close this gap. Through a collaboration between the Max Planck Institute for the History of Science and the Technical University of Berlin (Institute of Vocational Education and Work Studies), Sanctorius’s weighing chair was reconstructed and experiments carried out with it. This opened new perspectives on Sanctorius’s work and led to a reconsideration of the function and purpose of his weighing chair. With his *static medicine*, Sanctorius repurposed an old instrument. The replication of the weighing chair and the repetition of the experiments demonstrate that this novel application of scales posed some challenges for the mechanical design of the instrument. We recognized that the instrument fulfilled different functions that might in turn have affected its design, precision, and the measuring method applied. Although in the end we could not clarify how Sanctorius actually conducted his measurements, we were nevertheless able to develop an understanding of Sanctorius’s mechanical and practical knowledge that would not have been possible for us to develop solely on the basis of the written sources.

## Zusammenfassung

Im Jahr 1614 veröffentlichte der Arzt Sanctorius Sanctorius (1561–1636) sein berühmtestes Werk mit dem Titel *Ars […] de statica medicina* (*Über die statische Medizin*). Es handelt sich dabei um eine in Aphorismen verfasste Arbeit, die theoretische Betrachtungen zugunsten einer praktischen Orientierung in den Hintergrund drängt. So ist *De statica medicina* das Ergebnis einer größeren Anzahl von Messreihen, die Sanctorius mit einer selbst konstruierten Stuhlwaage über viele Jahre hinweg durchführte, um die sogenannte *perspiratio insensibilis*, eine unmerkliche Ausdünstung des menschlichen Körpers, quantitativ zu erfassen. Mit seinen Gewichtsexperimenten führte Sanctorius die Idee einer quantitativen Forschung in die Physiologie ein. Obwohl Sanctorius in historischen Darstellungen eine bedeutende Rolle als Begründer einer neuen medizinischen Wissenschaft zukommt, wurden die Konstruktion seiner Stuhlwaage und das Messverfahren bisher nicht näher analysiert. Der vorliegende Beitrag soll diese Lücke schließen. Im Rahmen einer Kooperation des Max-Planck-Instituts für Wissenschaftsgeschichte und der Technischen Universität Berlin (Institut für Berufliche Bildung und Arbeitslehre) wurde Sanctorius Stuhlwaage rekonstruiert und Experimente damit durchgeführt. Dies eröffnete eine neue Perspektive auf Sanctorius Unternehmungen und führte dazu, die Funktion und Verwendung seiner Stuhlwaage zu überdenken. Mit der statischen Medizin ordnete Sanctorius einem bereits lang bekannten Instrument einen neuen Zweck zu. Das Replizieren der Stuhlwaage und das Nachstellen der Experimente zeigten uns, dass diese neue Verwendung der Waage einige Herausforderungen an ihre mechanische Ausführung stellen. Wir erkannten, dass das Instrument verschiedene Funktionen erfüllte und dies möglicherweise wiederum Auswirkungen auf seine Konstruktion, Präzision und die angewandte Messmethode hatte. Auch wenn wir nicht abschließend klären können, wie Sanctorius seine Messungen tatsächlich umsetzte, so gelang es uns dennoch, ein Verständnis für das mechanische und praktische Wissen von Sanctorius zu entwickeln, wie es uns auf Basis der schriftlichen Quellen alleine nicht möglich gewesen wäre.

The use of balances is very normal to us today. We use kitchen scales to bake cakes, weigh birthday parcels in order to calculate the postal charges, and try desperately not to exceed the weight limits for our luggage before heading to the airport. To keep track of our own weight, most of us own bathroom scales, which have become more and more precise and can now even determine the percentage of body fat or muscle mass in a human body.

In effect, the balance is one of the oldest measuring instruments. Most probably, it was invented during the Neolithic period, when commerce and trade routes began to develop and the first towns were established (Robens et al. 2014: ix). Despite the early emergence of balances and weighing practices, it was only at the turn of the seventeenth century that they were applied to the human body. During this time, the physician Sanctorius Sanctorius (1561–1636) devised a weighing chair to undertake a series of experiments to measure and quantify physiological processes.^1^ As trivial as quantitative assessment with regard to health issues might seem to us today, it was a highly innovative step at the time. By experimenting with the weighing chair, Sanctorius introduced quantitative research into physiology.

Historical accounts of Sanctorius and his work tend to present him as a genial outsider, who, almost out of the blue, invented a new medical science that profoundly influenced modernity.^2^ This new science is identified as either iatrophysics, iatromechanics, or sometimes iatromathematics. These terms by no means constitute clear categories; rather, they are flexible appellations that have been applied retrospectively to developments in medical and natural philosophical research. However, the terms are comparable because they all reflect the importance of measurement and quantification in medical research, as well as the tendency to utilize numerical values and mechanical aspects in the field.

Given the important role that Sanctorius holds in these accounts, it is striking that the published reception of his ideas in the seventeenth and eighteenth centuries and the historical accounts of his work lack a detailed discussion of the design of his weighing chair and of the measuring method he applied to the weighing experiments. The aim of this paper is to address this gap. It presents the historical results of an ongoing research project on Sanctorius’s work and on the emergence of quantitative physiological reasoning at the turn of the seventeenth century. This project is being undertaken at the Max Planck Institute for the History of Science. In collaboration with the workshops of the Technical University of Berlin (the Institute of Vocational Education and Work Studies), we reconstructed the weighing chair of Sanctorius and conducted a series of experiments with it, partly undertaken in the framework of a seminar at the History of Science Department at the Technical University Berlin.^3^ This pilot study allowed us to develop a new perspective on Sanctorius’s undertakings and made us reconsider the function and purpose of the Sanctorian chair. It serves as the starting point for the research we continue to conduct toward a full analysis of the design of the weighing chair and of Sanctorius’s applied measuring method.

In what follows, I will present the current state of our results in three steps. First, I will analyze the primary sources on Sanctorius and his weighing chair in order to understand the instrument and the weighing process; I will also describe their reception and interpretation. Second, I will give an account of our reconstruction of and experiments with the Sanctorian weighing chair. Finally, I will summarize the conclusions we have drawn from these two lines of research. In practice, these elements were of course not separate from each other but rather closely intertwined and often mutually stimulating. The continuous rereading of the sources, combined with the knowledge that we gradually gained by reconstructing and conducting experiments with the Sanctorian weighing chair, led us to a new understanding of Sanctorius’s static medicine.

## Sanctorius’s *De statica medicina*

In 1614, Sanctorius Sanctorius published the book entitled *Ars […] de statica medicina *in Venice (Sanctorius 1614). It is a relatively short work, composed in the form of aphorisms that present the results of his weighing experiments. According to Sanctorius, the constant supervision of bodily discharges was essential for the preservation of health. In keeping with the classical view, he conceived of health as an ideal balance between ingestion and excretion, meaning that the quantity of food ingested should be proportionate to the amount of liquids discharged by the body (Dacome 2001: 472). The measurements Sanctorius conducted with the weighing chair demonstrated that a large part of excretion takes place invisibly through the skin and lungs (Grmek 1975: 102). Referring to the medical authorities of Hippocrates and Galen, Sanctorius conceived of this so-called *perspiratio insensibilis *as an imperceptible excretion of moisture through which the body rids itself of harmful and polluting matter through the pores of the skin (Dacome 2001: 427). It became the focus of the *De statica medicina*:

It is a new and unheard-of thing in Medicine that anyone should be able to arrive at an exact measurement of insensible perspiration. Nor has anyone either Philosopher or Physician dared to attack this part of medical inquiry (Sanctorius 1614: Ad lectorem).^4^

In Sanctorius’s view, the monitoring of the *perspiratio insensibilis* by means of systematic weighing was fundamental for the preservation of health. How did Sanctorius manage to weigh the *perspiratio insensibilis *precisely? To address this question, I will now take a closer look at the actual protagonist of the *De statica medicina*—the weighing chair Sanctorius designed in order to conduct his procedures.

## The Sanctorian Chair

**Fig. 1:**
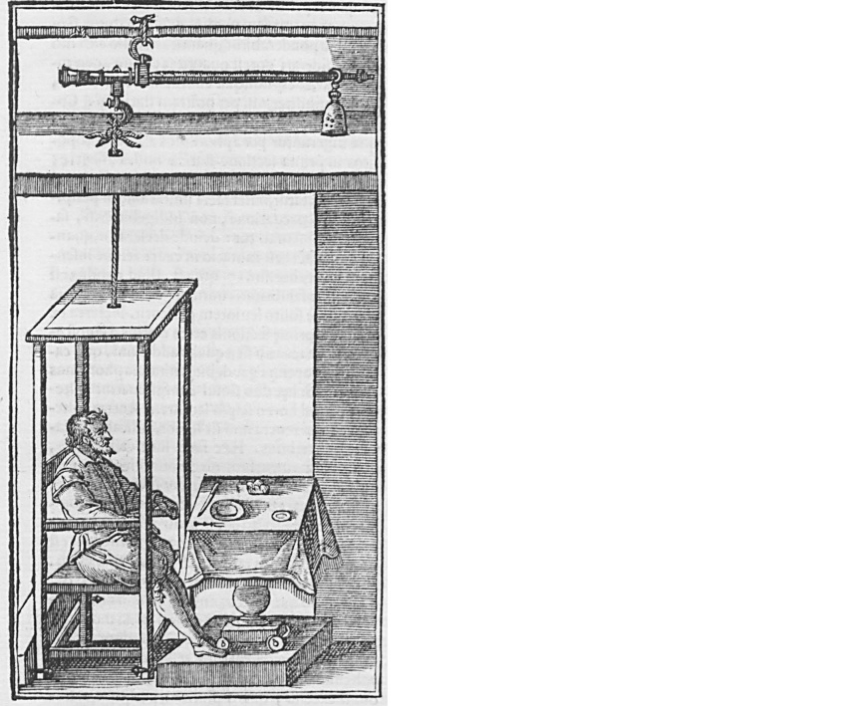
The Original Illustration of the Sanctorian Chair. From: (Sanctorius 1625: 557). Source: © British Library Board 542.h.11

*Statica medicina* is a practice of medicine based on weighing and thus on the use of a balance or a steelyard (Santorio & Ongaro 2001: 30). Interestingly, Sanctorius described and illustrated his weighing chair (together with other instruments he devised) in a commentary on the *Canon of Avicenna* (*Commentaria in primam fen primi libri Canonis Avicennae*) that was published in 1625—more than ten years after the *De statica medicina*:

The proposed aphorisms and those that are contained in our book of statics […] are proven true by the use of this chair, from which we draw two advantages. First, how much *perspiratio insensibilis* of our bodies occurs daily: which, if not rightly weighed, renders medicine altogether vain. For nearly all bad illnesses usually originate from a smaller or larger perspiration than is proper. Secondly, sitting in this chair and easily eating in between, we observe when we reach the due quantity of food and drink in excess of which or in shortage of which we are injured. The chair is arranged as it appears in the figure (see Fig. 1), in which the steelyard is suspended from the beams above the dining room, in a hidden place because of the nobles, as it renders the room less appealing, and because of the ignoramuses, to whom all unusual things appear ridiculous. The chair remains lifted from the floor at a finger’s height, stable in such a way that it cannot be easily moved; when, due to the ingested food, one reaches the expected weight and the measure previously set, then the outermost part of the balance ascends a little and contemporaneously the chair descends a little. This descent immediately indicates to the sitter that he has arrived at the stabilized quantity of food; which quantity or weight of salutary food is advisable for somebody, and how high the insensible transpiration in the individual bodies should be, one weighs comfortably with the chair. […] (Sanctorius 1625: 557 f.).^5^

Hence, the Sanctorian chair consisted of a chair hanging from one of the beams of a large steelyard and was meant to monitor bodily losses by means of systematic weighing. These losses indicated the quantities of sensible and insensible excretions and allowed Sanctorius to define a healthy quantity of the *perspiratio insensibilis*. The other purpose of the weighing chair was to determine the healthy amount of food for each person using the chair to eat. Before a meal, one had to set a measure correspondent to the quantity of food one wanted to ingest. During the weighing procedure, the weighing chair would drop. As soon as one had reached the set measure, the meal would end. According to Sanctorius, the healthy amount of food was directly connected to the quantity of the *perspiratio insensibilis*, as the quantity, quality, and type of food and drink affected the expulsion and retention of the sensible and insensible excretions. Moreover, both the quantity of food and drink and the quantities of sensible and insensible excretions were also influenced by other aspects, like the temperament of the person using the chair or the environmental and meteorological climate in which the person lived and in which the weighing took place. This reflects the dietetic concerns that were common in early modern medical thinking and practice.

The *De statica medicina* was organized according to the concept of the six *res non-naturales* (the six non-naturals), which constitute the main determinants of health and disease in traditional dietetic medicine (Jarcho 1970: 374). The six *res non-naturales* are categories of factors to which human beings are unavoidably exposed in the course of daily life and that influence health or disease, depending on the circumstances of their use or abuse. Generally, they are classified into the following categories: (1) air, (2) food and drink, (3) sleep and wakefulness, (4) motion and rest, (5) evacuation and repletion, (6) passions of the mind. Management of the patient’s regimen (that is, of these six sets of factors) was for centuries the physician’s most important task (Rather 1968: 337). In the *De statica medicina* there is a slightly adapted list of the non-naturals, which forms six of the seven sections into which the work is divided: *De Aere & aquis* (air and water), *De Cibo & potu* (food and drink), *De Somno & vigilia* (sleep and wakefulness), *De Exercitio & quiete* (exercise and rest), *De Venere* (coitus), *De Animi affectibus* (affections of the mind). These are sections II to VII. Section I is entitled *De ponderatione insensibilis perspirationis* and deals with the method of weighing insensible perspiration (Sanctorius 1614: Index). Thus, Sanctorius used a common concept of dietetic medicine to structure his work with the weighing chair, but shifted the focus to the *perspiratio insensibilis* and to the effect the non-naturals have on its excretion.

One can only speculate why Sanctorius did not add an illustration and a description of the instrument to the original editions of the *De statica medicina* even though its content is so closely connected with it. He simply might not have felt the need to do so. However, after having finally added the illustration to the commentary on Avicenna, it was often reproduced in the later editions of the *De statica medicina* with some changes—changes that seem minor at first glance.^6^ To some of them a description was added, also taken from the commentary on Avicenna.^7^ Giuseppe Ongaro assumes in the introduction to his edition of the *De statica medicina* that the illustration contributed much to the success of the *De statica medicina *(Santorio & Ongaro 2001: 34). Lucia Dacome identifies it in her article as “an integral, non-verbal and crucial component of static medicine’s rhetorical apparatus (Dacome 2001: 475).”

## The Use of the Sanctorian Chair as Described in the Sources

No detailed records of Sanctorius’s static experiments have been found. It is therefore commonly assumed that he did not leave any. Nevertheless, we know that he conducted them over a long period of time. According to his own claim, Sanctorius observed more than ten thousand subjects over the course of around thirty years.^8^ If one believes Sanctorius’s statements, he must have conducted the experiments constantly, as he writes in the preface to the *De statica medicina*: “[…] the same experiments, in which I was daily engaged through continued studies for many years, […] (Sanctorius 1614: Ad lectorem).”^9^

Perusal of this work shows how carefully Sanctorius carried out his experiments. In one of the aphorisms, he gives quantities for the amount of excrements expelled in one night: 16 ounces of urine and four ounces of stool. This number, together with knowledge of the quantity of the food previously ingested, enabled Sanctorius to determine the quantity of the *perspiratio insensibilis* that was expelled in one night. According to his measurements, it amounted to forty ounces or more (Sanctorius 1614: 13 v.).^10^ In addition to the evacuation of feces, urine, and *perspiratio insensibilis*, Sanctorius also refers to sweat, though in these cases he does not give exact quantities but remains more general (Sanctorius 1614: e. g., 4 r.; 5 v.; 10 r.; 14 r.–14 v.).^11^ Moreover, Sanctorius did not only weigh people before and after meals^12^, but at regular intervals during the day and night. Following the list of the six non-naturals, he tried to include parameters like climate, sleep, exercise, age, and even affections of the mind in his weighing experiments. In addition to the monitoring of variations in the *perspiratio insensibilis*, Sanctorius also tried to regulate these variations in order to establish the parameters of an ideal balance between ingestion and excretion:

How much is necessary for every one to perspire, in order to preserve a State of perfect Health, may be thus known. Take notice, in a Morning, following a plentiful Supper, of the greatest Quantity that perspires in the space of twelve Hours. Suppose it be fifty Ounces: Some other Morning observe the same, after eating no Supper, (and provided there was no Excess in the former Day’s Dinner) which suppose to be twenty Ounces: Then chuse such a settled Quantity of Food, and keep to such a use of the Non naturals, as will bring the Quantity perspired to a Mean between fifty and twenty Ounces, which is thirty five Ounces; and by this means may a Person be brought to such a perfect Standard of Health as will last to a Hundred Years (Santorio & Quincy 1712: 34).^13^

A few aphorisms show that Sanctorius also observed the absolute weight of individuals using the chair (Sanctorius 1614: 25 r.; 47 v.). In this context he defines a healthy weight range between 200 *libbre* and 205 *libbre* (Sanctorius 1614: 18 v.). We can assume that this weight range refers to adults, as the unit of *libbra* was equivalent to approximately one-third of a kilogram (Santorio & Ongaro 2001: 46).

Given the scarce information Sanctorius left us regarding his experimental setup and the experiments themselves, one might imagine that his brief description of the weighing chair together with the illustration would have given rise to many different interpretations. Indeed, some authors (among them Giuseppe Ongaro in his study of 2001) have felt the need to highlight that there was only a chair—and not a table or a bed, as others claim—hanging from the steelyard (Santorio & Ongaro 2001: 34; Ettari & Procopio 1968: 64). However, there seems to be a general consensus on the overall functioning of the weighing chair, and there is little or no discussion at all with regard to the exact design or the measuring method Sanctorius used.^14^ The outline of Sanctorius’s static medicine presented in the three preceding sections sketches this common discussion in the secondary literature.^15^ Taking this as a starting point, we set out to reconstruct the Sanctorian chair. Things soon began to look different, as I will show.

## The Reconstruction of the Sanctorian Chair

We used the replication method to develop a deeper understanding of the mechanical knowledge involved in the *De statica medicina*. This approach can be summarized in three stages: reconstruction of the apparatus, replication of the experiments, and contextualization of the experience gained in the first two stages.^16^

Without discussing this methodology in detail, some aspects of how we applied it to Sanctorius’s experiments must be mentioned to explain its potential for understanding the practical aspects of the weighing experiments. Our motivation for using the replication method was not to check the historical results but to develop an understanding of the practice. We wanted to build a functioning instrument with the technical potential and tools of today. This approach is not a full historical replication and must therefore proceed with a clear self-reflexiveness regarding its modern methods (Heering 2010: 796; Heering 2008: 350, fn. 15). Its focus is not on the historical details of how the balance was produced, but rather of how it possibly could have been used. Thus, when we staged the experiments on the basis of the information provided in the source material, we tried to develop a deeper understanding of the experimental procedures and the skills involved in performing them. Simultaneously, we reflected on our own practices with the instrument and how these practices developed over the course of the project.

In order to reconstruct an instrument of this size, we needed a workshop as well as professional support. We found both at the Institute of Vocational Education and Work Studies of the Technical University of Berlin. The head of the wood workshop, Katharina Wegener, not only gave us permission to use the facilities, but also greatly supported us in the realization of the project. A trained carpenter with a BA in History of Science, she was the ideal collaboration partner. Together with Matteo Valleriani, an expert in ancient and early modern mechanics, and Jochen Büttner, an expert in historical balances, we developed a design plan for the weighing chair on the basis of the original source material.

The illustration of the weighing chair indicates that Sanctorius used a Roman steelyard. Scales of this type were in wide use at the time, especially in a trading place like Venice, where Sanctorius began his weighing experiments. Steelyards the size of the Sanctorian chair were used to weigh sacks of flour or other commodities. Thus, we can assume that Sanctorius used a prefabricated steelyard for his weighing chair. The Roman steelyard consists of a straight beam with arms of unequal length. The beam is suspended from a defined pivot, which is flanked by the two arms. The longer arm is graduated and incorporates a counterweight, which can be moved along the arm to counterbalance the object to be weighed (the load) hanging on the short arm. When the two arms are balanced in a horizontal position about the pivot, the weight of the load is indicated by the position of the counterweight on the graduated arm. Thus, the weight can be either read directly from the graduation marks or calculated according to the law of the lever (Robens et al. 2014: 169).

**Fig. 2:**
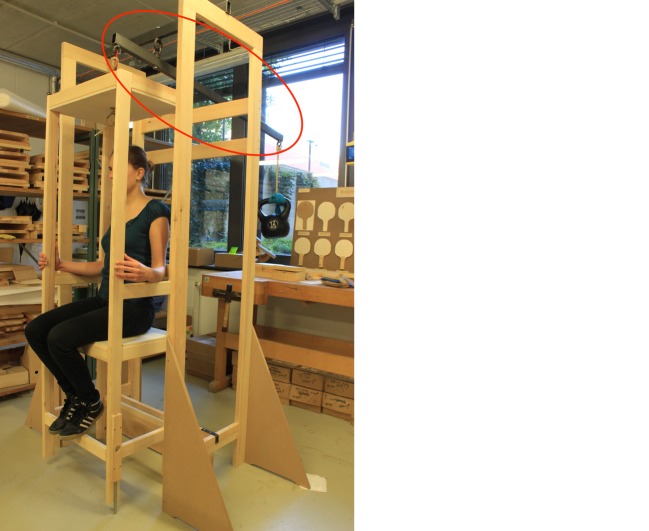
The First Reconstruction of the Sanctorian Chair. Source: © Philip Scupin

In contrast to Sanctorius, who suspended his weighing chair from the ceiling, we had to construct a stable framework in order to make our replication mobile (see Fig. 2).^17^ Therefore, we had to calculate measurements that guaranteed a manageable size. At the same time, we had to make sure that the chair could be used by people of varying weights. We used a beam with a length of 1.5 meters and defined a maximum load of 100 kg, including the weight of the chair. To keep the counterweight as light as possible, we decided to work with a proportion of 1:5, which corresponds to a counterweight of 20 kg for a load of 100 kg. This resulted in the following lengths of the arms that flank the pivot: a short arm of 25 cm, and a long arm of 1.25 m. We used structural steel for the beam and the pivot, and timber for the chair and the framework. After many hours of work in the wood workshop, and with the support of Volker Klohe, the head of the metal workshop, we finished a prototype with which we could begin experimenting (see Fig. 2).

But this is only half of the story.

## The Measuring Method

At first we assumed that Sanctorius used his model of a Roman steelyard in the traditional way described above. But during our ongoing discussion, we recognized two difficulties. Firstly, Sanctorius writes very clearly in his description of the weighing chair that a certain measure, which is set before the weighing starts, can be determined from the descent of the chair that is the distance of the chair to the floor. This indicates that the weight of the load is not read from the position of the counterweight hanging from the beam of the steelyard. Secondly, the actual steelyard was hidden behind the ceiling above the dining room. Thus, the arms of the beam and the counterweight were very difficult to access. Given the fact that Sanctorius used the weighing chair to monitor metabolic changes in many individuals of varying weights, he would have to balance the arms of the steelyard by moving the counterweight for every individual sitting on the chair anew—if he used the steelyard in the common way.

Based on these considerations, we again took a look at the original illustration of the Sanctorian chair. This time we specifically examined the lower part of the weighing chair. We could clearly identify little pointers or pegs at the bottom of the chair, attached to each leg. What were they intended for? Did they point to a scale that indicated to the sitter when he had reached the proper weight? Were they used to add weights to the sitter? Or did they serve to stabilize the suspended chair and prevent it from swinging? It is striking that this detail varies in later reproductions of the original illustration and that the variation has never been discussed. In the following, I shall briefly refer to one of the reproductions: the frontispiece of a Dutch edition of the *De statica medicina*, written by the physician Heidentryk Overkamp and published posthumously as part of his *Opera Omnia* (Overkamp 1694).^18^

**Fig. 3:**
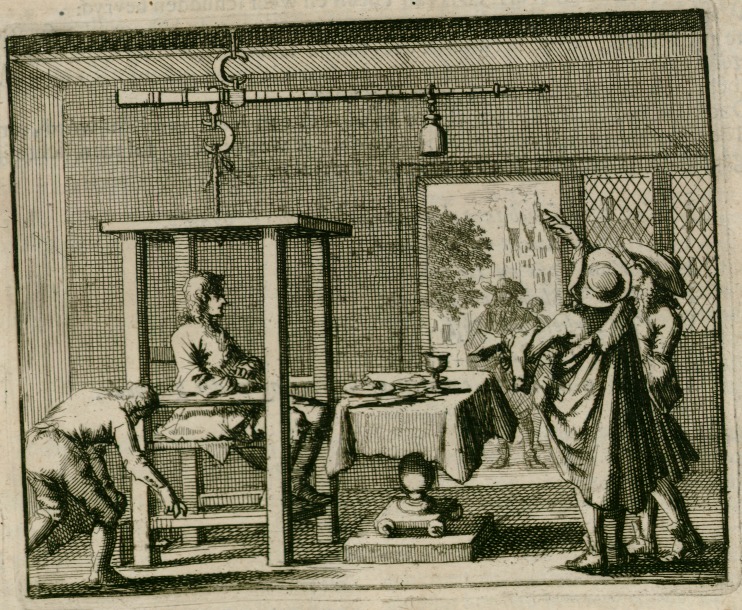
An Illustration of the Sanctorian Chair. From the Frontispiece to (Overkamp 1694). Source: Courtesy of Niedersächsische Staats- und Universitätsbibliothek Göttingen (SUB Göttingen)

The frontispiece shows a version of the Sanctorian chair (see Fig. 3) in which one can clearly identify one little pointer or peg at the right rear chair leg. Moreover, in contrast to the original illustration, it shows not only the person sitting on the weighing chair; three other people are displayed in the room in which the weighing experiments take place. The two people at the right seem to discuss the beam of the steelyard, the part of the weighing chair that is hidden behind the ceiling in the original. On the left, another person leans forward to the lower part of the chair, close to the point where the pointer or peg is placed. One cannot deduce with certainty the purpose of the pointer or peg on the basis of this illustration. Further, we do not know whether the person leaning forward is a craftsman, a servant, or a spectator interested in the weighing process. We do know that this person did not remove feces from the chair, as the weighing chair was not designed to be used as a lavatory. In Sanctorius’s response to a harsh critique by Hippolytus Obicius, a physician and philosopher of Ferrara, who attacked the *De statica medicina* violently in his work* Staticomastix sive Staticae Medicinae demolitio* (Obicius 1615), Sanctorius defends himself against the accusation of the inappropriate practice of weighing feces with the weighing chair (Sanctorius 1634: 69 r.).^19^

Even though many questions remain open, it has therefore become apparent that the lower part of the chair and its descent are important with regard to the weighing procedures, and most probably for the measuring method as well. It was interpreted in the reception of Sanctorius’s *De statica medicina* in different ways, but has never been included in a historical reconstruction until the present day. With this in mind, we started the experiments with our replica.

## Experimenting with the Reconstruction

**Fig. 4:**
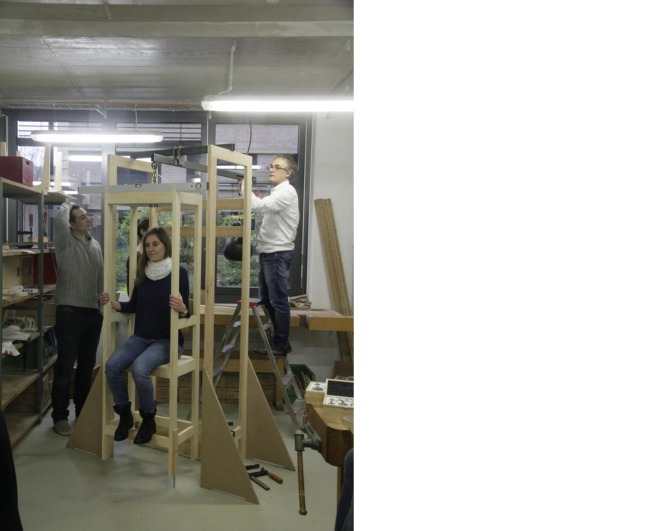
The Adapted Reconstruction of the Sanctorian Chair. Source: © Paul Weisflog

The experimentation process can be divided into two phases. In the first phase we used the prototype mentioned above (see Fig. 2) with two people of differing weights. In a second phase we experimented with an adapted and improved version of the prototype, which we constructed on the basis of the experiences gained in the first phase (see Fig. 4). We did so in the context of a seminar that was held as part of the curriculum of the History of Science Department at the Technical University of Berlin.^20^ In this second series of experiments, seven different individuals used the chair.

We conducted the experiments in both phases over several hours on one day. The aims were to test the functioning and precision of our reconstruction, to analyze different possible measuring methods, and to define potential scales. Thereby we wanted to understand the mechanical knowledge involved in the weighing procedures. Moreover, in performing Sanctorius’s experiments ourselves, we aimed to develop a better understanding of their method. As the purpose and use of the weighing chair are closely connected to its design, these objectives could not be analyzed separately but had to be considered as complimentary to each other. In the following, I will give a brief overview of the two series of experiments and present the conclusions that we drew from them.

Before the actual weighing can begin, a starting point has to be defined. This point guarantees the universal validity of the measuring process, with universal means valid for everyone, independent of their individual constitutions. As Sanctorius weighed many individuals of varying weights, he had to make sure that the beam of his weighing chair was balanced for the person sitting on the chair before he could start experimenting. There is good reason to assume that this starting point should be the balanced, horizontal position of the beam about the pivot—this is a common reference point—but it might be any position as long as it is the same for everyone using the chair. Most probably there was a marking somewhere at the bottom of the chair that indicated when the chair had arrived at the starting point. There are various ways to define this point. The most obvious is to use the steelyard in the classical way by moving the counterweight attached to the beam above the ceiling. This would have to be done for every person anew. Another method, not as obvious but far more comfortable, is to add weights to the person sitting on the weighing chair to compensate for the differences in weight. Thus, the beam of the steelyard is balanced once for a rather heavy weight and further weights are added when necessary, but this time to the load (that is, to the chair). In this method, the counterweight does not have to be moved for every individual to reach the starting point. This enables balancing the weighing chair without climbing up to the ceiling over the dining room.^21^

In addition to the starting point, there has to be at least one other marking at the bottom of the chair. As mentioned above, Sanctorius refers in his description of the weighing chair to a certain measure that is set before the weighing starts and indicates to the sitter when he has ingested the sufficient amount of food and drink. Sanctorius explicitly states that the quantity of ingested food and drink is indicated by the *descent* of the chair. Thus, he used the weighing chair not only to observe weight loss but weight gain as well. Where this second marking has to be—meaning the mark for the quantity of food and drink Sanctorius advises individuals to ingest—remains vague to the modern reader. Sanctorius not only connects it with the amount of excreted insensible perspiration, but also refers to the six non-naturals, whereby he includes a variety of parameters that influence the quantity of food and drink that an individual person should ingest. This leads to the conclusion that Sanctorius based the position of the second marking on contemporary dietetic knowledge and the experiences he gained during the weighing procedures.

So when we tried to define this second marking in our experiments with the reconstruction, we did not deal with an exact quantity but rather attempted to determine how a certain quantity (the second marking) could generally be determined for different individuals using the chair. Here again, different possibilities can be found through different methods of determining the starting point. If we balance the beam by moving the counterweight hanging from the steelyard above the ceiling and we do this for every individual with regard to their weight, the descent of the chair is proportional to the weight of the load. Therefore, a universal marking could be attached to the bottom of the chair, indicating when each individual person has ingested the sufficient amount of food and drink. Another option is to determine the weight of a person by the steelyard in the common way and then use the same method again to set a desired weight for the food and drink that a person should ingest. The counterweight could be moved to such a position that once the previously set measure is reached the chair not only drops down but touches the ground. Using the ground floor in this way as an indicator of when the desired amount of food and drink has been ingested is of course also possible for the method mentioned before, instead of setting a mark close to the bottom of the chair. If we use the other option and add weights to the person sitting on the chair to balance the beam of the steelyard, the amount of food ingested can be indicated by a graduated scale attached to the bottom of the chair. Here the initial weight of the load is the same for everyone using the chair. Thus, the descent of the chair after a meal is not proportional to the weight of the individual person. The addition or removal of weights to or from the chair might have enabled Sanctorius to identify exact quantities not only above the ceiling by looking at the position of the movable weight hanging from the beam of the steelyard, but also by looking at the bottom of the chair.

## Precision of the Reconstruction and of the Sanctorian Chair

**Fig. 5:**
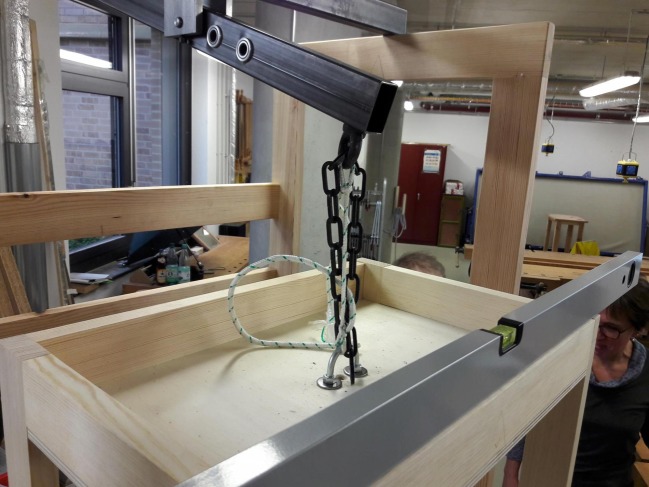
The Suspension of the Chair. Source: © Paul Weisflog

As soon as we started to include the descent of the chair in our procedures and tested its possible function as an indicator of changes in weight, flaws in our reconstruction came to light. It turned out that the chair was very unsteady and sensitive to any kind of movement. Thus, the various persons using the chair not only had to keep still during the measuring process, but also had to adopt an identical seating position. To prevent the chair from rotating to one side, we replaced the rope that suspended the chair in our first version of the prototype with a steel chain that we attached to the chair with the help of a U-bolt (see Fig. 5). Additionally, on the basis of the original illustration of the Sanctorian chair, we placed a wood panel behind our chair, attached to the framework (see Figs. 2 and 4). This also helped us to prevent major oscillations, even though we had to be careful to keep the friction between the wood panel and the chair legs to a minimum so as not to falsify the measurements. Even a small disequilibrium caused perceptible differences in the descent of the chair. This became even more obvious when we started to add weights to the person sitting on the chair. The added weights had to be distributed equally over the chair to prevent it from descending more on one side than on the other. A spirit level, attached to the top of the chair, helped us to monitor its horizontal position.

Our experiences showed that the suspension of the chair to the beam, as depicted in the original illustration, is prone to rotation and prevents a precise reading of measurements by means of the chair’s descent. However, Sanctorius was well aware of this difficulty, as he states in the description of the weighing chair: “the chair remains […] stable in such a way that it cannot be easily moved; […] (Sanctorius 1625: 558)”. Unfortunately, he does not reveal to the reader how he achieved stability. Thus, we can only speculate that he might have used the pegs that we identified at each chair leg for stabilization. Arranged between the wood panel behind the chair and a platform holding the dining table, the pegs might have served as guidance to keep the chair as steady as possible. Based on these considerations, the idea evolved to add stable guides to the chair legs, which we plan to implement in a future version of our reconstruction.

**Fig. 6:**
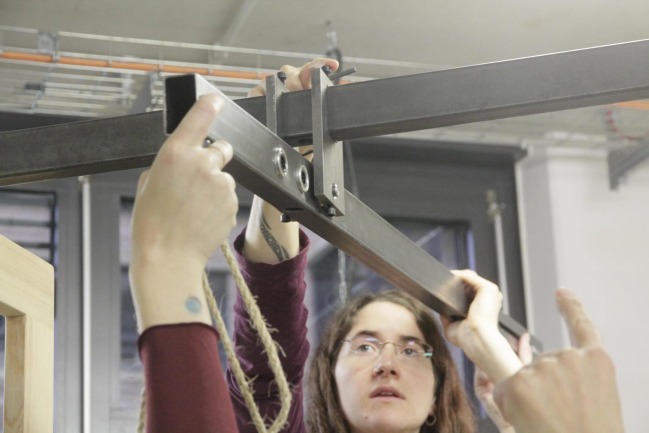
The Ball Bearing to Minimize the Distance between the Pivot and the Lever. Source: © Paul Weisflog

When we tried to define the second marking at the bottom of the chair, we realized that its descent was not proportional to the weight of the load. Moreover, only large differences in weight had an effect on the descent of the chair. As the figure shows (see Fig. 2), in the first version of our reconstruction the pivot is located between two steel rings that are welded together and form the fulcrum, which is attached to the stable framework and substitutes for the ceiling. In order to make our weighing chair more precise we had to minimize the distance between the pivot and the lever. However, we had to be careful to find the right distance, as minimizing the distance between the pivot and the lever not only makes the steelyard more precise but simultaneously causes smaller inclinations of the beam, which makes it more difficult to determine minor differences in weight. Hence, we had to find a solution that on the one hand guaranteed the necessary precision of the weighing chair and on the other hand still allowed us to read the measurements at the bottom. We solved this problem with the modern solution of a ball bearing (see Fig. 6). Sanctorius, of course, had to find another method. The original illustration of the weighing chair shows that he connected the lever directly to the hook on the ceiling with some kind of box or rectangular guide, which made the distance between the pivot and the lever relatively small.

The precision of a steelyard also depends on the length of the beam. To adapt this parameter to our needs in relation to the different persons using the chair and to guarantee the highest possible precision, we replaced the initial suspension hook with three hooks at different positions on the beam of our prototype (see Fig. 6). This resulted in the following lengths of the arms flanking the pivot. First hook: short arm 17.5 cm; long arm 1.325 m. Second hook: short arm 23.5 cm; long arm 1.265 m. Third hook: short arm 29.5 cm; long arm 1.205 m. Our experiments with the different hooks showed that the third hook, the one closest to the end, was ideal for our load weight range of 66 kg to 75 kg when working with a movable counterweight of 20 kg.

On the basis of the original illustration of the Sanctorian chair, we can assume that Sanctorius used a beam with a length of around 3 m—twice as long as the beam in our reconstruction. This enabled him to achieve high precision in his measurements and to reduce the counterweight. In a future version of our reconstruction, we also plan to extend the beam to allow for a greater weight range, to allow for the use of a handier counterweight of less than 20 kg, and to further enhance precision. Here again the right lengths have to be found in order to keep the steelyard precise and the measurements discernible. Sanctorius might have equipped his weighing chair with different hooks, too, even though the illustration does not clearly indicate this. Steelyards with up to three suspension hooks had been in use for weighing objects of varying weights since the Roman Empire (Robens et al. 2014: 169).

The adapted and improved version of our prototype with regard to the oscillation of the chair, the distance between the pivot and the lever, and the length of the beam allowed us to measure differences in weight by means of the descent of the chair with a precision of up to 100 g in the second series of experiments. This comes close to the precision that Sanctorius claims to have measured in the *De statica medicina*. The minimum quantity to which Sanctorius refers in his aphorisms is four ounces, which corresponds to around 100 g if we calculate with the Venetian *oncia sottile*. In the aphorism mentioned above (see sect. “The Sanctorian Chair”), Sanctorius states that up to 16 ounces of urine are usually expelled in one night. In several other aphorisms, especially of the third section, *Food and Drink*, he gives quantities of six, twelve, 14, 18, and 22 ounces. He writes for example: “Very nourishing Meats, excepting *Mutton* [*sic*] between the time of Supper and Dinner, do not Perspire above Eighteen Ounces (Santorio & Quincy 1712: 112).”^22^ This indicates that he worked with a steelyard that had a precision of one ounce. This in itself is nothing out of the ordinary: at the time, steelyards were used to weigh loads ranging from ounces to tons. But merchants and traders who had to weigh small, ounce-sized merchandise usually used small, portable steelyards of only some ten centimeters in length (Robens et al. 2014: 169). In contrast, steelyards of the size of Sanctorius’s weighing chair were commonly used to weigh sacks or barrels of commodities in which precision to the ounce was hardly needed. Thus, the mechanical challenge of the Sanctorian chair is to develop a design that on one hand allows the weighing of heavy loads up to around 80 or 90 kg, and on the other guarantees high precision to note even minor variations in weight.

## Reading of Measurements

We developed and tested various methods for reading the measurements. We added markings to the beam of the weighing chair indicating the respective starting points of the varying persons using the chair. This was relatively easy and only became difficult when we tried to discern differences in weight. Calibration of the longer arm requires skill and great accuracy. Since we worked with a counterweight of 20 kg, it was extremely difficult to record minor weight differences, which corresponded to only very short lengths of the beam. Sanctorius probably did not face these problems as we can assume that he worked with a calibrated steelyard, which were used widely at the time.

**Fig. 7:**
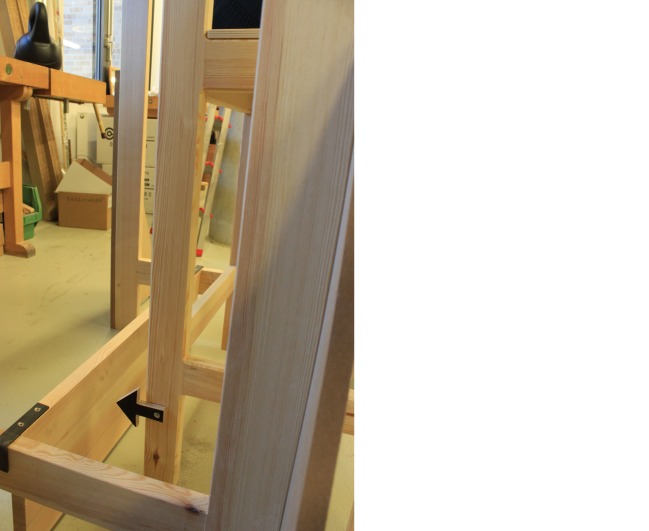
Wooden Arrow as Indicator for the Descent of the Weighing Chair. Source: © Philip Scupin

**Fig. 8:**
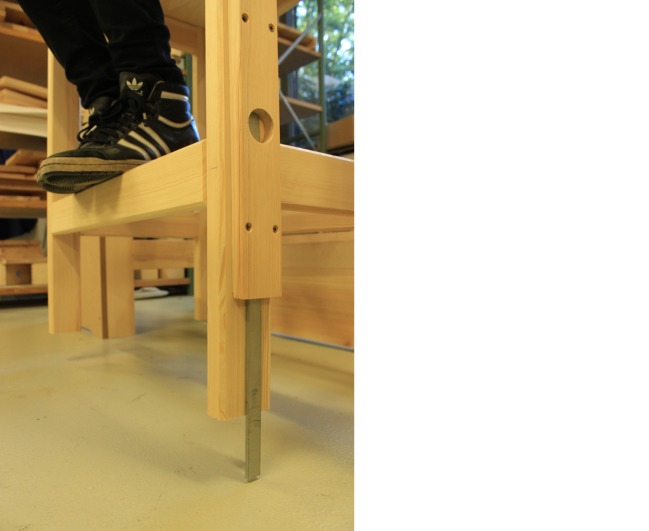
Steel Bar as Indicator for the Descent of the Weighing Chair. Source: © Philip Scupin

To measure and mark the descent of the chair, we developed different solutions that we tested in our experiments. The figure (see Fig. 7) shows that we attached a wooden arrow to one leg of the weighing chair. Touching the wood panel behind the chair, it was meant to indicate the chair’s descent. Inspired by another reproduction of the illustration of the Sanctorian chair, we attached a steel bar in a loose wooden duct to a different leg. The steel bar touches the ground and is held upright by means of the loose duct attached to the leg of the chair; as the chair moves up and down, the steel bar indicates these movements (see Fig. 8).^23^ Our experiments showed that the use of the arrow to indicate the descent of the chair was problematic. We applied reference points to the wood panel to which the arrow points, but reading the measurements this way was very difficult. Even though we had already enhanced the stability of the chair in our second prototype, the person seated on the chair still had to remain in a very stable, balanced position to prevent the chair from descending more on one side than on the other. For every measurement, the distribution of the load on the chair had to be identical. The steel bar is far easier to handle and allows a very exact reading of the measurements. As the figure indicates, a graded scale is still missing. However, we plan to exchange the bar with a steel ruler in a future version of the reconstruction.

On the basis of the original illustration of the Sanctorian chair that depicts pointers or pegs attached to each chair leg, we can assume that these might have served as indicators of the descent of the chair, similar to the arrow that we used in our experiments. However, we cannot deduce this with certainty. As mentioned above, they might also have served as stabilization. Further, there is the possibility of a combined function. The two pointers or pegs at the rear end might have served as fixed guides to ensure stability, while the ones at the front end served as indicators of the descent of the chair, pointing to the platform on which the table is placed. There is no evidence that Sanctorius used a steel bar as an indicator, since it appears only in a later reproduction of the Sanctorian chair, which also differs slightly from the one we used in our experiments. We applied the steel bar to our reconstruction to investigate different possibilities for measuring the descent of the chair.

## Measuring Parameters

In addition to the functioning of our reconstruction and the analysis of the different measuring methods, we also had to consider the different parameters that Sanctorius included in his weighing procedures. As already mentioned, the structure of the *De statica medicina* was influenced by the concept of the six *res non-naturales*, which reflects Sanctorius’s conviction that the quantities of sensible and insensible excretions were influenced by all of these factors. Thus, our central question was: What was Sanctorius actually able to determine quantitatively? This is not easy to answer, as Sanctorius presented his results mostly in the form of qualitative aphorisms rather than quantitative tables. Perusal of the aphorisms shows that he strongly adhered to Galenic medicine, the medical authority of the time. He complemented his measurements with qualitative conclusions and general observations that he made during the weighing experiments and integrated them into Galenic theory.

Interestingly, Sanctorius only specifies precise quantities in the first four sections of the *De statica medicina*, whereas he confines himself to more general and rather qualitative statements in sections V to VII. In some cases, we can draw a connection between the mention of precise quantities and Sanctorius’s ability (or inability) to measure certain parameters, as for example in the last section, *De Animi affectibus* (affections of the mind). Here, Sanctorius demonstrates how rage, joy, fear, melancholia, and consolation affect perspiration: “Amongst the Affections of the Mind, those of Anger and Joy, make Persons lighter, those of Fear and Sorrow, more heavy; and the other Affections operate in Proportion to their Participation of these (Santorio & Quincy 1712: 263).”^24^ Thus, Sanctorius states quite generally that some passions provoke weight loss whereas others provoke weight gain. We can assume that it was extremely difficult for him to specify precise quantities of weight gain or weight loss directly connected to the passions of the many different individuals he observed on his weighing chair. This becomes even more obvious when reenacting the experiments. It is often hard to tell what emotions you have or what mood you are in. For the chair to measure such things, as soon as you recognized a mood change, you would have to use the weighing chair to determine how this was affecting your weight and the excretion of the *perspiratio insensibilis*. In contrast to the affections of the mind, the third section, *De Cibo & potu* (food and drink), is far easier to apply to the weighing procedures. The quantities of ingested food and drink can be controlled and monitored relatively easily. This might also be the reason why Sanctorius specifies many precise quantities in this section.

The aim of our experiments was not to verify the exact results that Sanctorius presents in the *De statica medicina*, but to develop a general understanding of their method. Our experiments showed that our reconstruction needs to be further adapted to allow for a full analysis of Sanctorius’s weighing procedures. Systematic long-term measurements, including all of the parameters that Sanctorius mentions, are planned. However, in this pilot phase we tried to reenact the weighing procedures as much as possible. We created tables for every person that used the chair to record their age and mood, the time and date, and the weather before the measurements were taken. Moreover, they had to indicate whether they moved or rested before the weighing took place, whether they slept or were awake, and whether they consumed food and drink. After the measurements had been performed, they had to calculate how much food they ingested, how much they excreted insensibly, and how much they excreted through feces. Thus, we had to weigh them at regular intervals during the duration of the experiments, immediately before and after eating, and immediately before and after going to the toilet. In keeping with our interpretation of Sanctorius’s description of the device (see fn. 12), we did not allow a person to eat whilst in the chair. At this stage, the calculations were intended to give us a general idea of how Sanctorius’s weighing practice might have looked; they did not provide reliable data to develop a scale or to verify Sanctorius’s measurements. Nevertheless, these experiments made us understand how difficult it is to include all the various parameters in the measurements and to consider them in conjunction with one another. This is a difficulty that Sanctorius faced himself. In his aphorisms, he only ever determined a selection of the interactions between the many parameters and their impact on the physiology of human beings. There is no coherent list of parameters that he takes into account in every measurement. Hence, apart from the mechanical challenges of the weighing chair, a comprehensive imitation of Sanctorius’s procedures requires careful preparation and a close reading of the aphorisms of the *De statica medicina*. It demands a high level of self-discipline and a regular and uniform lifestyle, always within reach of the weighing chair.^25^

## The Sanctorian Chair: A Multi-functional Instrument?

With his weighing chair, Sanctorius repurposed an old instrument. Our reassessment of the original source materials based on the experience gained through reconstructing the Sanctorian chair and replicating the weighing experiments taught us how this novel application of the steelyard raises challenges for the mechanical design of the instrument. It also widened our perspective to the great variety of its applications. Different measuring methods can be applied that directly affect the design, the functioning, and the precision of the weighing chair. Although our research does not allow us to unambiguously define the measuring method Sanctorius used, it has shown that this method is not as self-evident as was commonly assumed and that it warrants further investigation. Systematic long-term measurements with a further-adapted version of the reconstruction have to be undertaken.

On the basis of our research, we can assume that Sanctorius most likely used some variation of the measuring methods mentioned above. He used both the steelyard concealed behind the ceiling and at least two reference points attached to the bottom of the chair. Even though the original illustration of the weighing chair gives no clear indication of a scale at the bottom of the chair or on the wood panel behind it, scaling would have been necessary to these two reference points. In short, Sanctorius had to translate weight into a distance. He thus worked with proportions as well as with exact quantities. Whether the pointers or pegs at the bottom of the chair served as indicators for these reference points, whether they were meant to stabilize the chair, or whether they fulfilled both functions requires further investigation.

The aphorisms of the *De statica medicina* and the description of the Sanctorian chair imply that the instrument had two functions. On the one hand, it was used as a research tool to monitor variations in the production of *perspiratio insensibilis*; on the other, it helped to determine and maintain an ideal body weight. The measuring methods might have varied in correspondence with these two functions. Based on our experiences with the reconstruction, it seems likely that Sanctorius used the steelyard in the traditional way, especially in the initial phase of his experiments, when he tried to define the healthy quantity of insensible perspiration. As soon as he managed to stabilize this quantity, he could determine the ideal body weight for different individuals and determine the healthy amount of food and drink that they should ingest. To this purpose, he might have used the descent of the chair as an indication of changes in weight, as described in the commentary on Avicenna. This would have enabled individuals, even laymen, to use the chair on their own, without the need of an assistant to move the counterweight along the longer arm of the weighing chair. In this regard, the weighing chair would not have been meant for use by multiple individuals, but only by one person; the beam of the steelyard would therefore be balanced only once, for that person’s respective weight. This fits with Sanctorius’s suggestion that the beam of the steelyard be hidden above the ceiling to avoid the astonishment of guests, to whom the weighing device would look ridiculous. It implies that Sanctorius may have conceived of the chair for a larger public to regulate their eating habits.^26^

In this context, it is important to keep in mind that Sanctorius published the description and illustration of the Sanctorian chair eleven years after the *De statica medicina*. This could reflect the development of his research during these years. After beginning with the aim to determine the quantity of the *perspiratio insensibilis* within the frame of contemporary dietetic medicine, he might have realized that the chair not only helped the physician to monitor changes in weight and, on this basis, to issue rules of health, but also offered the opportunity to find and maintain an ideal weight. In order to make the chair accessible to laymen, he might have adapted the design and measuring method with regard to this newly discovered function and published both in his commentary on Avicenna.^27^

Over the course of the eighteenth century, the two functions of the Sanctorian chair were hotly debated (Dacome 2001: 475). Who should use the Sanctorian chair? Was it designed for medical or lay practice? With the primary sources at hand, we still cannot unambiguously answer these questions. However, the methodological approach of the replication enabled us to find a possible connection between the different functions of the Sanctorian chair and its design and measuring methods. During our research, we developed a new understanding of the mechanical and practical knowledge involved in Sanctorius’s weighing procedures—an understanding that we would have hardly developed on the basis of the written sources alone.

## Acknowledgements

I thank Friedrich Steinle, Hans-Liudger Dienel, and Jürgen Renn, who have rendered the collaboration between the Max Planck Institute for the History of Science and the Institute of Vocational Education and Work Studies (TU Berlin) possible. Friedrich Steinle provided invaluable comments that greatly improved the manuscript. I would like to thank Katharina Wegener and Volker Klohe, who supported me in the process of the replication; without their help, it would have been impossible to realize this project. I am also immensely grateful to Jochen Büttner, who provided insight and expertise that greatly assisted the research. I would also like to thank Lindy Divarci, Zachary Gresham, and Lindsay Parkhowell for their editorial work. Last but not least, I sincerely thank Matteo Valleriani for his ongoing support throughout the whole project and for his precious advice, comments, and critiques.

## Footnotes

1 Before Sanctorius, the philosopher and cardinal Nicolaus Cusanus (1401–1464) had already suggested the use of a balance for medical purposes (e. g., to weigh blood and urine). In the short treatise *De staticis experimentis *(Static experiments) Cusanus presents his innovative ideas. However, we can assume that Cusanus’s work remained on a speculative level and that his weighing experiments were only imaginary. Nevertheless, their impact, even as purely conceptual experiments, should not be underestimated. The influence of Cusanus on Sanctorius has been under debate since the publication of Sanctorius’s *De statica medicina*. An ongoing research project on Sanctorius’s work and on the emergence of quantitative physiological reasoning at the turn of the seventeenth century—undertaken at the Max Planck Institute for the History of Science—is currently reanalyzing this issue. For the previous debate, see Santorio and Ongaro (2001: 21 f.); Agrimi et al. (1996: 122–125). For an English translation of Cusanus’s *De staticis experimentis*, see Viets (1922).

2 See, for example, Ettari and Procopio (1968); Castiglioni (1931); Guidone and Zurlini (2002); Vedrani (1920); Giordano (1924); Major (1938).

3 For a detailed visual documentation of the reconstruction project, see: https://www.mpiwg-berlin.mpg.de/research/projects/reconstruction-sanctorian-chair. This web page was created thanks to the support of Stephanie Hood.

4 “Novum atque inauditum est in medicina posse quēpiā ad exactam perspirationis insensibilis ponderationem pervenire: nec quisquam Philosophorum, nec Medicorum unquam hanc medicae facultatis particulam aggredi ausus est: […].” See Sanctorius (1614: Ad Lectorem). For the English translation, see Foster (1924: 145).

5 “Propositi aphorismi, & illi, qui continentur in libro staticae nostrae aliquot iam per annos in lucem edito, veritate comprobantur ex usu istius sellae: ex qua duo beneficia colligimus. Primum quanta quotidiè fiat corporis nostri perspiratio insensibilis: qua non rectè perpensa, vana fermè redditur medicina: namq; ob iusto pauciorē, vel largiorem perspirationem omnes ferè malae valetudines fieri solent. Secundum, in hac sella sedendo facilè intercomedendū animadvertimus, quando pervenimus ad debitam cibi & potus quantitatem, ultra vel citra quam, laedimur. Sella accommodatur, sicuti in hac figura apparet, in qua statera ad tigna supra caenaculum in loco ab. dito est appensa propter proceres, quia cubiculi gratiā tollit, ac propter indoctos, quibus omnia insolita videntur ridicula: Sella verò digiti interstitio à pavimento elevata manet, stabilis, ne facilè quassari possit: dum igitur ob cibū ingestū ad debitum pondus, & mensuram antea praescriptā devenimus: tunc staterae extrema pars paululum attollitur, ac una sella illicò paululum descendit: Hic descensus est ille, qui statim admonet sedentem ad debitam ciborum quantitatem pervenisse: quaenam verò ciborum salubrium. quantitas seu pondus unicuique conveniat: & quanta in singulis corporibus debeat esse perspiratio insensibilis quae per sellam commodè perpenditur, ex lib. nostro de statica medicina quisque facilè intelliget.” See Sanctorius (1625: 557 f.). English translation by the author of the present work, with the help of the Italian translation according to Santorio and Ongaro (2001: 33).

6 For the epistemic function of images in scientific texts, see Valleriani et al. (2017). This work examines the systematic implementation of a pictorial program in scientific texts in Western and Latin cultures from the beginning of the fourteenth century. For an investigation of the processes of codification of practical activities and practical knowledge, see Valleriani (2017).

7 The descriptions of the weighing chair in the various editions of the *De statica medicina* seem to have remained unchanged and are thus identical with the description in the commentary on Avicenna. See, for example, Sanctorius (1660).

8 In the preface of the *De statica medicina*, Sanctorius states that he conducted the experiments over the course of thirty years. See Sanctorius (1614: Ad lectorem). However, in a letter Sanctorius sent to Galileo Galilei with a copy of his *De statica medicina* in 1615, he mentions that he carried out the experiments over a span of 25 years. See Sanctorius (1902).

9 “[…] quandoquidem ipsa experimenta, quibus quotidie assiduis multorum annorum studiis incumbebam […].” See Sanctorius (1614: Ad lectorem). English translation by the author of the present work.

10 The unit of measurement and of weight used by Sanctorius—the *libra* (pound)—was variable between the different Italian states. The most common was the Roman *libra*, which was equal to 327.45 g and divided into twelve *unciae* (ounces: one ounce = 27.2875 g). In Venice, the *oncia grossa* (39.5 g) and the *oncia sottile* (25 g) were in use. We do not know to which *oncia* Sanctorius refers in the aphorisms of the *De statica medicina*. In the introduction to his edition of the *De statica medicina*, Giuseppe Ongaro refers to the *oncia sottile* without explaining the choice. Given the precision of Sanctorius’s quantitative statements and his aim to quantify the *perspiratio insensibilis*, this choice seems reasonable to the author of the present work. See Santorio and Ongaro (2001: 46).

11 For an analysis of early modern medical concepts of sweat, see Stolberg (2012). In this paper, Stolberg refers to the relationship between sweating and insensible perspiration, which was controversially discussed in early modern writings about sweat. An extensive analysis of Sanctorius’s concept of sweat and *perspiratio insensibilis* is part of the ongoing research project on Sanctorius’s work and the emergence of quantitative physiological reasoning at the turn of the seventeenth century. This research is being undertaken at the Max Planck Institute for the History of Science.

12 In the original Latin description of the weighing chair, Sanctorius writes “[…] in hac sella sedendo facilè intercomedendū […].” See Sanctorius (1625: 557 f.). See also the English translation in sect. “The Sanctorian Chair”. The Latin preposition *inter* can be translated as either *between* or *during*. In connection with the verb *comedere*, the author of the present work considers the translation *between* to be more accurate.

13 “Quanta conveniat perspiratio cuilibet, ut conservetur in statu saluberrimo, sic dignosces. Observa manè post aliquam pleniorem caenam illam maiorem perspirationem, quae in teipso duodecim horarū spatio fieri possit: esto esse quinquaginta uncias: alio mane; sed poste ieiunium, hac tamen lege, ne in prandio praeteritae diei excesseris, idem observa; ponamus esse viginti; hoc praecognito, eligas illam cibi, & aliarum causarum non naturalium moderationem, quae te ad medium inter quinquaginta & viginti quotidie ducere poterit; medium erit triginta quinque unciarum; hoc modo sanissimam, & diutissimam seù centum annorum vitam duces.” See Sanctorius (1614: 14 v.–15 v.). For the English translation, see Santorio & Quincy (1712: 34).

14 As Lucia Dacome points out in her article (Dacome 2001), many scholars performed Sanctorius’s weighing experiments well into the eighteenth century in France, Britain, Ireland, Colonial America (South Carolina), and the Netherlands. However, these imitators did not prioritize the historical accuracy of Sanctorius’s experiments, but were rather interested in his novel idea and method of quantification. To them, the output was more important than the design and measuring method Sanctorius used. Thus, they left detailed static tables that indicate their commitment to drawing general conclusions concerning the relationship between intake, weight, and health based on minute calculations of bodily excretions. Most of them did not even describe their experimental setup. Hence we do not know which balances they used for their re-trials, whether they tried to reconstruct the original Sanctorian chair, or if they invented novel constructions. One exception is Jacob Leupold (1674–1727), who not only described in detail his own design of a weighing chair, but also criticized the design illustrated in the *De statica medicina* and even stated that this design cannot have been used in the way Sanctorius described in his commentary on Avicenna. See Leupold (1726: 63).

15 See, for example, Dacome (2001); Santorio and Ongaro (2001); Ettari and Procopio (1968); Guidone and Zurlini (2002); Miessen (1940).

16 The replication method is an attempt to analyze historical experimental practice, as applied systematically by members of the Oldenburg Group. This group was established in Oldenburg in 1987 under the direction of Falk Rieß. See Heering (2008: 350, fn. 15). For an extensive study of the replication method and what the authors call “experimental history of science,” see Breidbach et al. (2010).

17 We had to make our replication transportable for two reasons. First, the limited space in the wood workshop where we reconstructed the Sanctorian chair did not allow for a permanent installation of the instrument. Second, we plan to exhibit the reconstruction in different locales on different occasions.

18 My thanks to Ruben Verwaal, who drew my attention to this illustration of the Sanctorian chair.

19 “Staticus scit pondus faecum, licet eas nec videat, nec perpendat. Corpus ante perpendit & iterum post omnem excretionem: quod deficit est earum pondus: Sic non est indgnum perpendere faeces, ut ait trico.” See Sanctorius (1634: 69 r.). In the secondary literature, it is also sometimes erroneously stated that Sanctorius weighed feces by means of a balance. See, for example, Major (1938: 374); Poma (2012: 215).

20 My thanks to Agnes Bauer, Nana Citron, Andrea Grimm, Alev Güzel, Leon Kokkoliadis, and Paul Weisflog for their active participation and the many fruitful discussions.

21 At the beginning of the eighteenth century, Jacob Leupold designed a portable weighing chair, the *machina antropometrica*, to which he applied a similar measuring method. In his work *Theatrum Staticum Universale* (1726), Leupold describes how the person using the *machina* should determine the counterweight on the basis of an estimate of his own body weight. According to Leupold, this estimate does not have to be accurate, as there are additional weights that the sitter puts on the arms of the chair to compensate for inaccuracies and to balance the two arms in a horizontal position about the pivot. See Leupold (1726: 64–66; table XVIII).

22 “Cibi multum nutrientes, excepta carne vervecina, à caena ad prandium non solēt perspirare ultrà octodecim uncias.” See Sanctorius (1614: 32 v.). For further examples, see ibid.: 32 r.; 39 r.–39 v.; 40 r.–40v.

23 For the illustration, see Beugo (2017).

24 “Inter affectus animi, ira & pericharia corpora efficiunt leviora: timor & maestitia graviora: caeteri vero affectus ut his participantes operantur.” See Sanctorius (1614: 75 r.). For the English translation, see Santorio & Quincy (1712: 263).

25 In the eighteenth century, the imitators of Sanctorius’s experiments already referred to the authority of the weighing chair and stated that the instrument imposed a certain degree of compulsion on the person performing the weighing procedures. See Dacome (2001: 478–480).

26 Lucia Dacome also points to the possibility that Sanctorius’s suggestion to hide the beam of the weighing chair above the ceiling implies that he may have conceived the chair for a larger public beyond the community of physicians. See ibid.: 476.

27 In their paper (Valleriani and Pearl 2017), Matteo Valleriani and Yifat-Sara Pearl refer to the use of images in teaching and learning and argue that the use of images in scientific texts lowers the threshold to knowledge and thereby renders knowledge accessible to wider audiences.
